# Boron Nanocomposites for Boron Neutron Capture Therapy and in Biomedicine: Evolvement and Challenges

**DOI:** 10.34133/bmr.0145

**Published:** 2025-02-25

**Authors:** Farooq Ahmad

**Affiliations:** State Key Laboratory of Chemistry and Utilization of Carbon Based Energy Resources, College of Chemistry, Xinjiang University, Urumqi 830017, China.

## Abstract

Cancer remains a major concern for human health worldwide. To fight the curse of cancer, boron neutron capture therapy is an incredibly advantageous modality in the treatment of cancer as compared to other radiotherapies. Due to tortuous vasculature in and around tumor regions, boron (^10^B) compounds preferentially house into tumor cells, creating a large dose gradient between the highly mingled cancer cells and normal cells. Epithermal or thermal neutron bombardment leads to tumor-cell-selective killing due to the generation of heavy particles yielded from in situ fission reaction. However, the major challenges for boron nanocomposites’ development have been from the synthesis part as well as the requirement for selective cancer targeting and the delivery of therapeutic concentrations of boron (^10^B) with nominal healthy tissue accumulation and retention. To circumvent the above challenges, this review discusses boride nanocomposite design, safety, and biocompatibility for biomedical applications for general public use. This review sparks interest in using boron nanocomposites as boron neutron capture therapy agents and repurposing them in comorbidity treatments, with future scientific challenges and opportunities, with a hope to accelerate the stimulus of developing possible boron composite nanomedicine research and applications worldwide.

## Introduction

Cancer is a substantial challenge for humanity. In attempts to carry out efficient cancer treatment, existing sophisticated cancer therapies, including surgery, chemotherapy, and radiation therapy, pose serious consequences due to nonselective killing of cancer cells as well as normal cells, especially in inoperable gliomas and head and neck cancers with higher chances of tumor recurrence and higher systemic chemotherapy and radiation toxicity. Therefore, to overcome the bottlenecks in glioblastoma multiforme (GBM) treatment, Sweet [1] from Massachusetts General Hospital suggested boron neutron capture therapy (BNCT) for GBM.

**Fig. 1.**
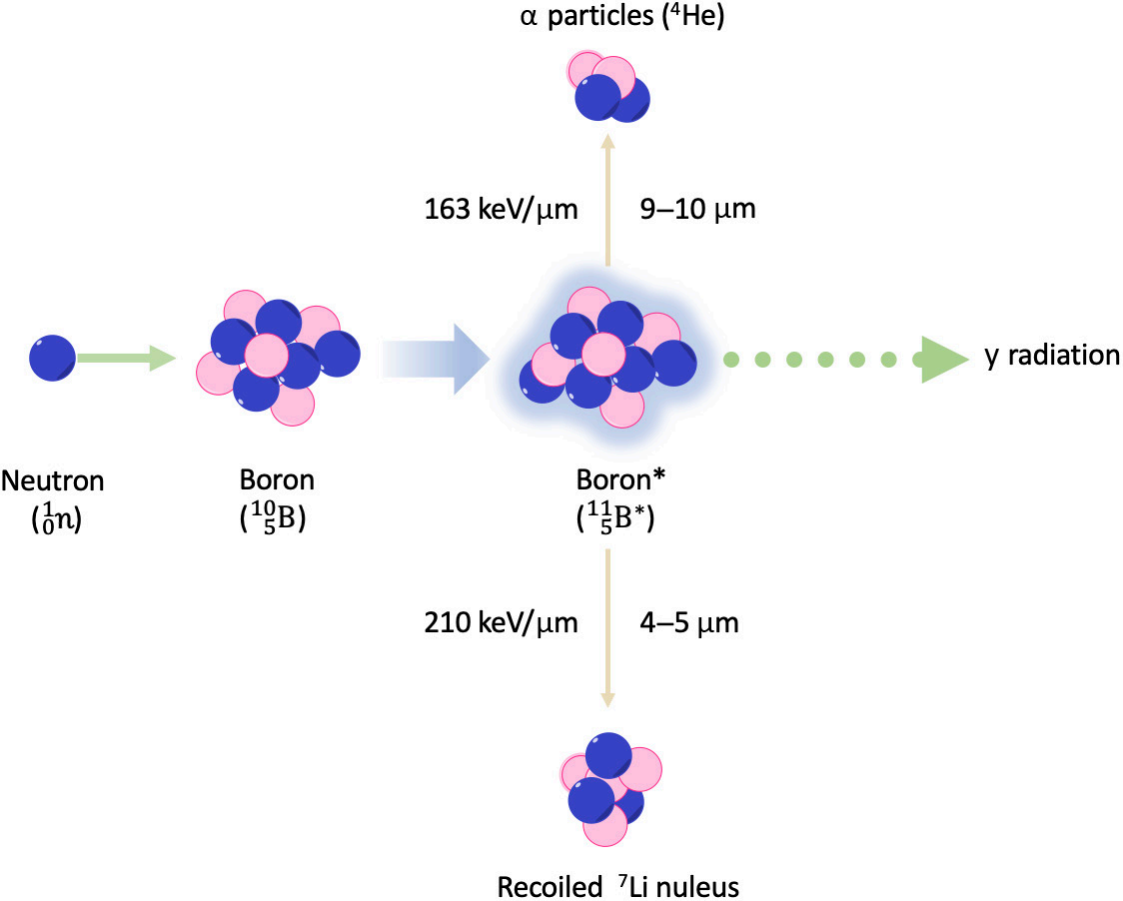
The principle of boron neutron capture therapy (BNCT).

BNCT is an advanced noninvasive binary targeting radiotherapeutic methodology for the targeted annihilation of deadly cancers [2]. BNCT eliminates the classic toxicities of radiotherapy and chemotherapy and offers treatment of nonoperable tumors such as head and neck cancers. This is due to its ability to kill neoplastic cells somewhat biochemically rather than by symmetrical targeting, and it is especially appropriate for treating infiltrating tumor cells, untraceable micrometastasis, the foci of malignant alteration in field-cancerized tissue, and radiosensitive organs such as the lungs or liver due to drug selectivity and physicochemical properties. Furthermore, BNCT provides fast, targeted treatment with 1 to 2 fractions of neutron irradiation, as opposed to the 30 fractions of conventional x-rays and proton therapy [3]. This potentially makes BNCT treatment easier and less costly and produce reduced radiotoxicity for patients and healthcare providers alike. BNCT involves the intravenous administration of ^10^B-containing medicine with targeted buildup into neoplastic cells [4]. Under neutron irradiation, only neoplastic cells containing ^10^B are killed by the boron neutron reaction through disintegration of ^10^B into high-energy alpha particles (^4^He) and ^7^Li recoil nuclei (Fig. 1). These high-energy transfer alpha particles (^4^He) and ^7^Li nuclei have a high linear energy transfer that is approximately within the size of the cell diameter (<10 μm) [5,6] and directly destroy the DNA double helix, mediating irreversible harms to neoplastic cells. Hence, only neoplastic cells containing ^10^B are killed by the boron neutron reaction, keeping the adjacent healthy cells intact. BNCT is therefore an ideal cell-targeting radiotherapy.

However, one of the major challenges in implementing BNCT at clinical settings is the use of a reactor-based neutron source, which cannot be installed at hospitals, and thus, only very limited people can be treated with BNCT worldwide. However, with technological advancements, scientists have developed portable accelerator-based neutron sources replacing the reactor-based neutron source, and these will soon be installed in hospitals, universities, or research institutes [7]. As of now, BNCT is being studied clinically in China and the USA; it has not yet received approval for general patient use. Japan is the only nation that has approved BNCT for clinical settings. In particular, brain tumors, malignant melanoma, osteosarcoma, and refractory, recurring, invasive, metastatic, and incurable malignancies have shown notable clinical success with BNCT and with boronophenylalanine (BPA) medication. For instance, in a phase II (JHN002) trial of a cyclotron-based epithermal neutron source, an intravenous borofalan (^10^B) dose of 400 mg/kg gives promising patient outcomes in patients with recurrent squamous cell carcinoma or with recurrent/locally advanced nonsquamous cell carcinoma of the head and neck [8]. However, the limitation of this study is that safety and efficacy assessment beyond 3 months was not conducted and it used a small pool of patients.

**Fig. 2.**
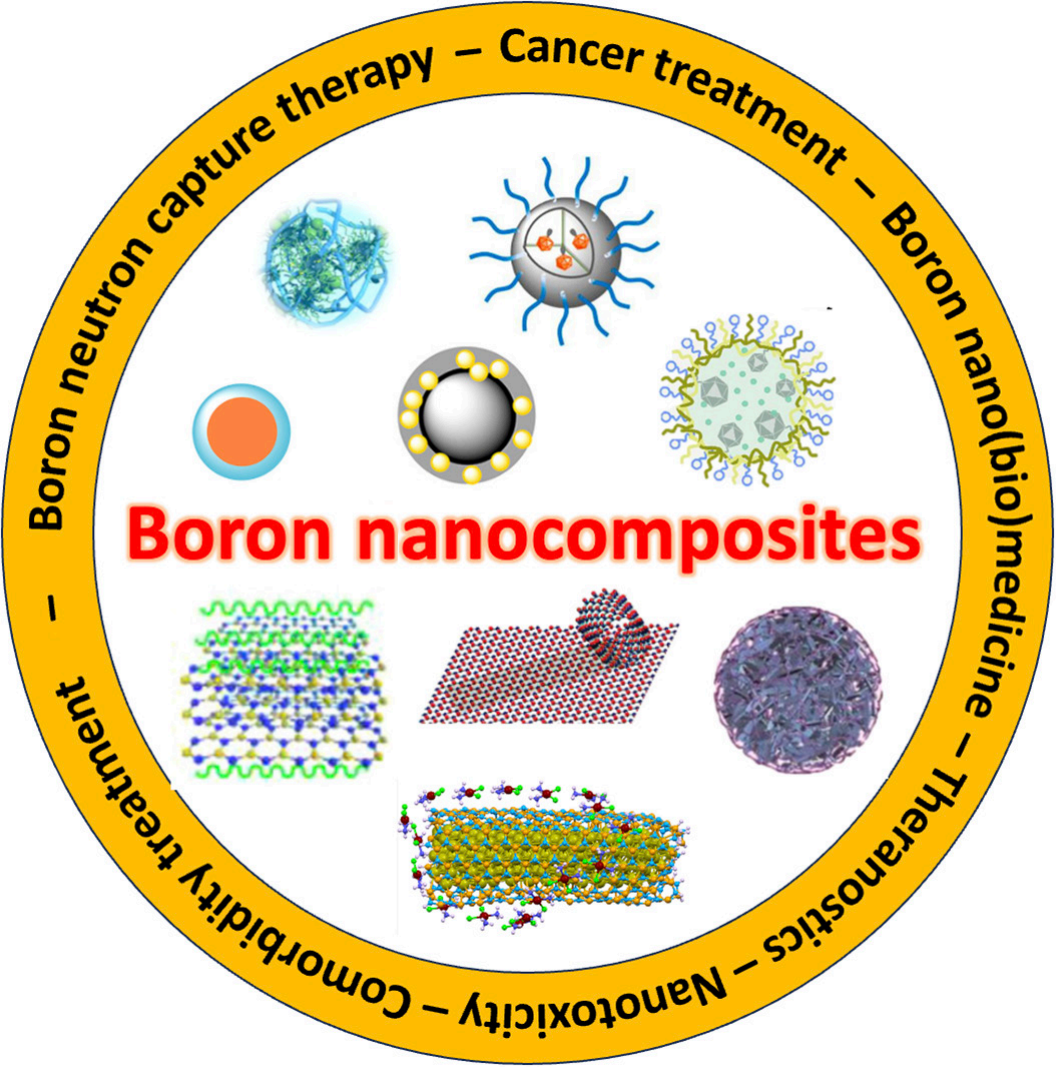
Overview of the review.

In this review, we first concisely discuss the advantages, issues, and problems of BNCT in general, specifically emphasizing the current state of boron agents, in particular the nanoparticles (NPs) and their pitfalls. It also includes the possible strategies to overcome the roadblocks in designing boron-containing theranostic nanoagents for wider biomedical applications. Finally, we also put emphasis on the application of boron nanoagents in the treatment of diseases (comorbidities) in combination with cancer treatment, providing multipronged biomedical applications, expanding the role of boron nanocomposites as novel nanomedicine (Fig. 2). Finally, we hope to spark new ideas and inspire new strategies to eliminate the roadblocks in providing cheaper and sustainable translation of novel boron-enriched nanostructures into clinical practice.

## Conventional Boron Agents for BNCT

Irrespective of the neutron source used, the therapeutic efficacy of BNCT depends on the boron abundance in the tumor area. There are only 2 well-established low-molecular-weight (LMW) boron compounds have been used in clinical trials: (a) sodium borocaptate (BSH) and (b) BPA. BSH was first used in clinics for BNCT treatment of brain tumor patients [9]. However, BPA was originally reported for clinical killing of melanoma [10] and later various cancers possessing an l-type amino acid transporter (LAT1) [11,12] followed by targeted treatment of brain tumors and head, neck, and lung cancers [13–15]. BPA proved to be efficient only in LAT1-expressing tumors, demanding the development of boron agents for targeting other kinds of tumors as well [16]. However, due to their low molecular weight and the relative concentration of amino acids across the cancer cell membrane, more than half of these ^10^B agents are rapidly cleared from the circulatory system within an hour of administration (*t*_1/2_ < 1 h). Therefore, to increase the tumor load of ^10^B up to 20 > 20 ppm concentration will be achieved with intravenous infusion [17,18]. This increases the nonspecific dispersion of LMW ^10^B agents throughout the entire body, leading to both systemic toxicity and adverse effects during thermal neutron irradiation [19,20].

Furthermore, during clinical trials, these LMW BSH and BPA also exhibited limitations of poor retention time and specific binding, respectively [21]. Therefore, to improve tumor retention, monosaccharides such as fructose and mannitol-based complexes of BPA were used, as they make boronated esters for enhanced water solubility in neutral pH and ultimately improved tumor retention [22]. Fructose-tailored poly(ethylene glycol) (PEG)–poly(l-lysine) strongly incorporating BPA (PEG–P[Lys/Lys(fructose)]) showed excellent in vitro and in vivo therapeutic outcomes. In addition, the PEG–P[Lys/Lys(fructose)]–BPA complex was efficiently internalized into cells through LAT1-mediated endocytosis and inhibited the untoward export by LAT1-facilitated renal clearance and increased the tumor/blood and tumor/normal brain ratios. This provides proof of concept that PEG–P[Lys/Lys(fructose)] and polymer–BPA combinations prolong the intertumoral retention and improve the physiochemistry of the polymer, critically affecting the biodistribution of polymer–BPA [23]. Nevertheless, this study did not provide information on how the boronated ester structure and binding constants affect the biological activity and why PEG–P[Lys/Lys(fructose)] exhibited higher affinity to BPA than fructose. Furthermore, in order to reduce the dependence on fructose or sorbitol used for boron formulation, researchers have synthesized a borylated amino acid equivalent of l-tyrosine called 4-borono-l-tyrosine (BTS). BTS used the same LAT1 family transport and tumor retention mechanisms [24]. This design is advantageous, leading to higher in vivo dosing, and has the potential to produce positive clinical BNCT outcomes. The above systems provide only one possible target site, and considering the heterogeneity of tumors, we need to develop boron agents with the ability to target multiple tumor progression routes/mechanisms irrespective of the heterogeneity of malignant tumors. Given these, these boron agents should show (a) high tumor selectivity, (b) low systemic toxicity, (c) a high concentration of boron in tissues of interest (~20 μg ^10^B/g tissue), (d) rapid blood and tissue clearance, and (e) retention of the boron composites in the tumor tissue [25]. No doubt, these requirements set a very high standard for the development of BNCT agents for clinical application, but they also provide the basic grounds for the discovery and development of future BNCT agents for general public use.

BPA and BSH have been produced at commercial scale by following good manufacturing practices (GMPs) for clinical application. However, next-generation BNCT agents are of urgent need to mitigate the disadvantages of BPA and BSH. Given that, various boron-containing agents or carrier (nano)systems have been designed by modification with amino acids [26], peptides [27], nucleosides [28], liposomes [29], antibodies [30], and many more [31]. However, in this review, our focus is on nanoscale boron agents or carriers.

## Boron Nanostructures as BNCT Agents

**Fig. 3.**
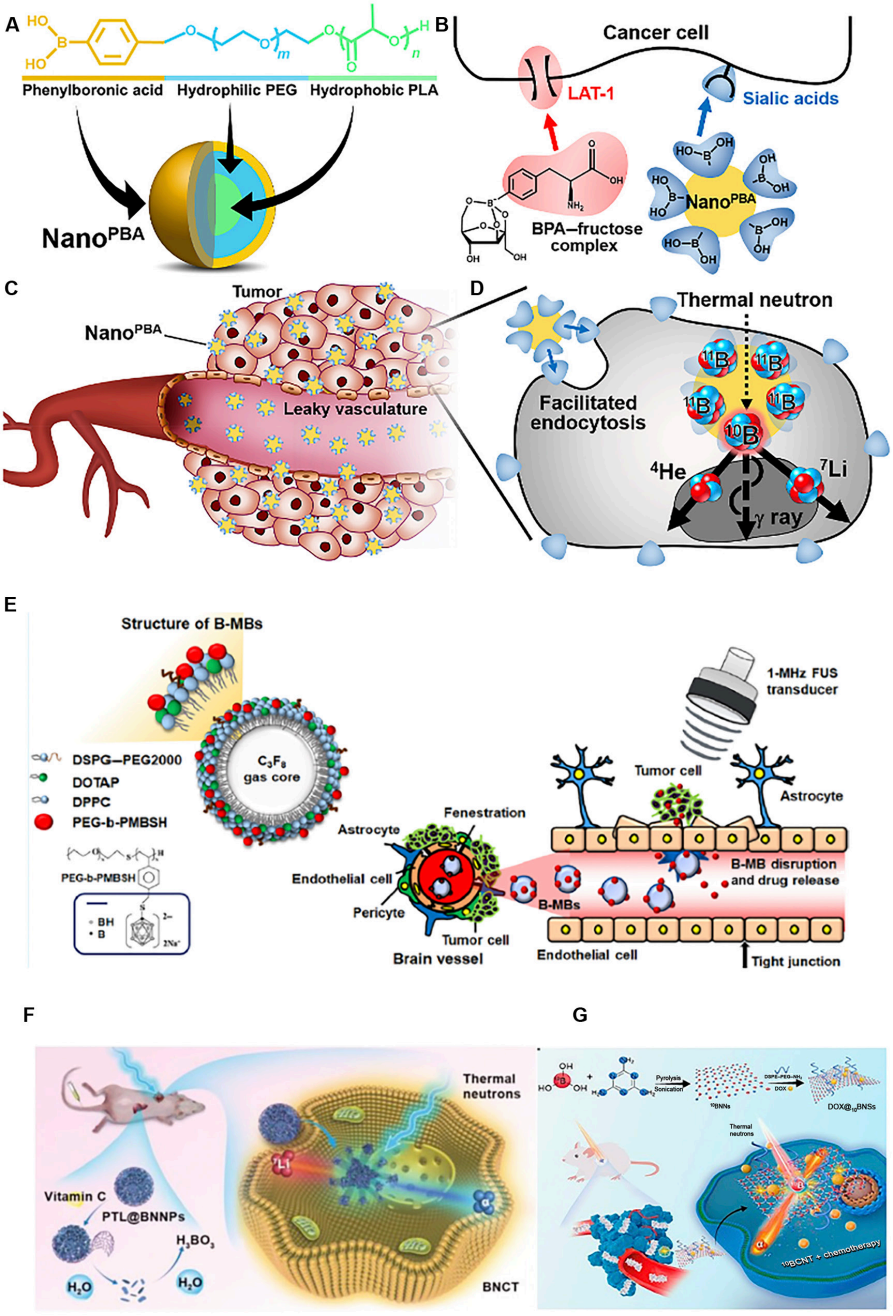
(A) The chemical structure and molecular design of sialic acid-linked phenylboronic acid polymeric nanoparticles (Nano^PBA^). (B) Distinct cellular uptake of the boronophenylalanine (BPA)–fructose complex compared to Nano^PBA^. (C) Passive tumor targeting via the enhanced permeability and retention (EPR) effect. (D) Facilitated endocytosis of Nano^PBA^ followed by nuclear fission in response to thermal neutron irradiation. Reproduced with permission from Ref. [34]. (E) Self-assembled sodium borocaptate polyanion (polyethylene glycol-β-poly((*closo*-dodecaboranyl)thiomethylstyrene)) nanoparticles coupled with cationic microbubbles (B-MBs) with focused-ultrasound (FUS)-enhanced boron drug delivery targeting brain tumor. Reproduced with permission from Ref. [35]. (F) PTL@BNNPs-based BNCT with on-demand degradation [38]. (G) DOX@BNNSs-based drug delivery for cancer radiochemotherapy. Reproduced with permission from Ref. [127]. PEG, poly(ethylene glycol); PLA, poly(lactic acid); LAT1, l-type amino acid transporter; BPA, boronophenylalanine; DSP–PEG2000, 1,2-distearoyl-*sn*-glycero-3-phosphoethanolamine-*N*-[methoxy(polyethylene glycol)-2000]; DOTAP, 1,2-dioleoyl-3-trimethylammonium propane; DPPC, dipalmitoylphosphatidylcholine; PEG-b-PMBSH, polyethylene glycol-b-poly((closo-dodecaboranyl)thiomethylstyrene; BH, borohydride (comes in sodium borohydride salt); PTL, phase-transitioned lysozyme; BNNPs, boron nitride nanoparticles; DOX, doxorubicin; BNNSs, ^10^B-rich nanosheets; PEG-b-PMBSH, polyethylene glycol-β-poly((*closo*-dodecaboranyl)thiomethylstyrene); DSPE, 1,2-distearoyl-*sn*-glycero-3-phosphorylethanolamine.

Owing to the astonishing physicochemical characteristics of nanomaterials, boron-based nanomaterials could enhance the bioaccumulation of ^10^B in the tumor site [2,4,5,19,32], due to poorly aligned neovascularization and the deficiency of effective lymphatic drainage through the so-called enhanced permeability and retention effect [33]. In addition, boron-based nano(bio)materials offer on-demand surface functionalization with biocompatible polymers, such as PEG, or target specific antibodies, exhibiting high colloidal stability along with nonbiofouling characteristics. This is advantageous in personalized boron-based nanomedicine development. For instance, researchers designed sialic acid-linked phenylboronic acid polymeric NPs as a sialic acid-targeting BNCT agent, with a core and a shell composed of poly(lactic acid) and PEG fragments, respectively (Fig. 3A to D).

Altogether, this system provides stronger multiple binding with the cancer cell membrane, leading to higher intracellular trafficking and thus requiring a 100-fold lower dose compared to naked BPA [34]. Self-assembled BSH polyanion (polyethylene glycol-β-poly((*closo*-dodecaboranyl)thiomethylstyrene)) NPs (295 ± 2.3 nm in aqueous media) coupled with cationic-microbubble-assisted focused ultrasound treatment (4 min) (Fig. 3E) lead to improved uptake of BSH in the brain, showing up to 3-fold improvements in the tumor/normal brain (4.4 ± 1.4 vs. 1.3 ± 0.1) and tumor/blood ratios (1.4 ± 0.6 vs. 0.1 ± 0.1), respectively [35]. Michiue et al. designed simple self-assembled A6K peptide nanotubes by mixing AK6 and BSH (AK6/BSH) in a 1:10 ratio with high intracellular transduction and nonspecific drug delivery to brain tumors (mouse model). The peptide-functionalized nanotubes improved BSH accumulation to be 10 times higher than that of BSH [5]. Conversely, one of the disadvantages was the complex synthesis steps, and the trickled drug integrated into the polymeric NPs during blood circulation [32,36,37]. Therefore, to overcome the challenges of undesired leakage of boron content from polymeric NPs, Li et al. designed an on-demand degradable boron carrier. Boron nitride nanoparticles (BNNPs) were coated by a phase-transitioned lysozyme that protected BNNPs from hydrolysis during blood circulation and were easily eliminated by vitamin C (Fig. 3F) after neutron capture therapy (NCT) [38]. The same research group also designed 2-dimensional ^10^B-rich nanosheets (BNNs), tailored as a multifunctional delivery system for BNCT and drug delivery vehicles to load doxorubicin for chemotherapy (Fig. 3G). Irradiated by low-energy thermal neutrons, BNNs produce high-linear-energy-transfer particles to kill tumor cells, and the loaded doxorubicin is released in situ at the same time.

No doubt, these NPs have a long circulation time, leading to higher tumor accumulation, but they are also packed with off-targeting problems, causing severe side effects such as bioaccumulation-mediated liver and spleen dysfunction, limiting their repeated administration. To overcome this problem, Feiner et al. used a pretargeting approach to tactfully control the tumor accumulation of synthesized boron-doped carbon dots (BCDs) surface functionalized with tetrazine. They showed fast clearance and low tumor accumulation in the mouse human epidermal growth factor receptor 2 (HER2) tumor model. However, after pretargeting of tumor with Food and Drug Administration-approved mononuclear antibody *trans*-cyclooctene-functionalized trastuzumab, they exhibited higher binding affinity for HER2 receptors. BCDs underwent biorthogonal reaction at the tumor site, leading to controlled accumulation in the tumor site without damaging vital organs [39]. However, there were also chances that this biorthogonal reaction was compromised due to biomolecular corona formation, limiting effective bioaccumulation into the tumor.

**Fig. 4.**
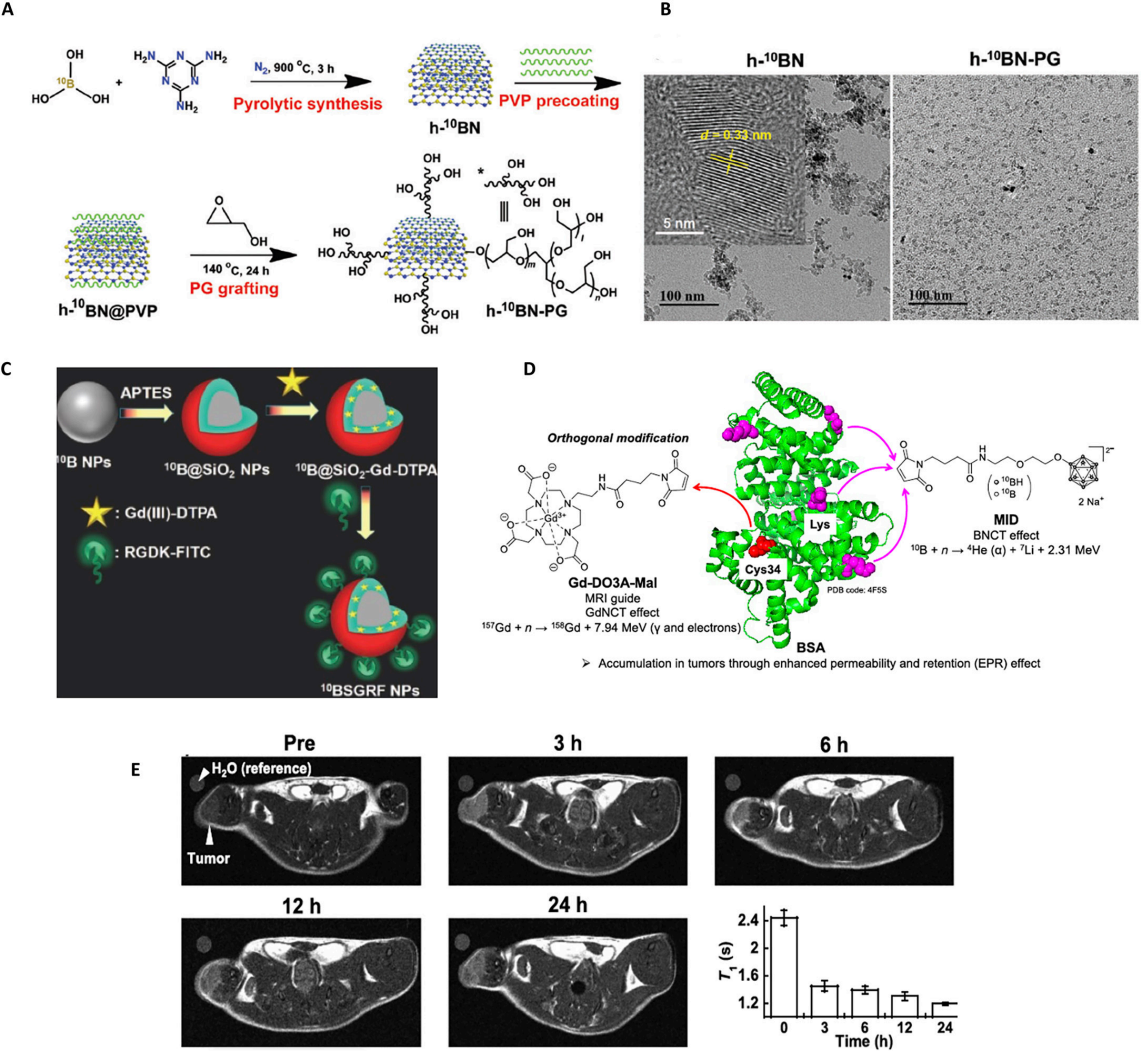
(A) Synthetic route of ^10^B-enriched hexagonal boron nitride nanoparticles grafted with polyglycerol (h-^10^BN-PG NPs). (B) Transmission electron microscopy (TEM) images of ^10^B-enriched hexagonal boron nitride (h-^10^BN) and h-^10^BN-PG NPs. Reproduced with permission from Ref. [43]. (C) Synthesis of ^10^B-enriched (96% ^10^B) nanoparticles (^10^BSGRF NPs) [62]. (D) Gadolinium–boron-conjugated albumin (Gd-MID-BSA). (E) Representative T_1_-weighted images of the tumor at 0 h and 3, 6, 12, and 24 h postdose and corresponding *T*_1_ time course of the tumor. Reproduced with permission from Ref. [75]. PVP, polyvinylpyrrolidone; PG, polyglycerol; APTES, (3-aminopropyl)triethoxysilane; DTPA, diethylenetriamine pentaacetate; FITC, fluorescein isothiocyanate; MRI, magnetic resonance imaging; PDB, Protein Data Bank; BSA, bovine serum albumin; Gd-DO3-Mal, maleimide-functionalized gadolinium complex; GdNCT, gadolinium neutron capture therapy; MID, maleimide-functionalized *closo*-dodecaborate albumin.

In a recent study, researchers synthesized a HER2-targeted antibody-attached boron nitride nanotube-β-1,3-glucan complex with a 30-fold increased therapeutic output compared to that of a clinically used l-BPA/fructose complex [40]. They developed the mechanochemical solubility technique to disperse the complex in water with the use of polysaccharides and β-glucans and their derivatives [41,42]. In a similar effort, ^10^B-enriched hexagonal boron nitride NPs grafted with polyglycerol (h-^10^BN-PG NPs) were synthesized as a robust and efficient ^10^B nanocarrier with 5-fold higher ^10^B loading and with hydrophilic and stealthy attributes by polyglycerol coating (Fig. 4A and B). Notably, the high tumor accumulation and small particle size of h-^10^BN-PG NPs supported deep penetration into the tumor parenchyma, boosting BNCT outcome with one-time neutron irradiation, even at a reduced radiation dose, without incorporation of any other adjuvant modalities such as chemo- and phototherapies. This work evidenced the activation of the tumor immune microenvironment by BNCT, eventually leading to significant tumor shrinkage [43]. Similarly, brown color elemental boron NPs (BNPs) were synthesized by pulsed laser ablation with a size distribution from 20 to 50 nm. Structural analysis revealed the formation of amorphous and partially crystalline BNPs NPs, functionalized with PEG polymers. In vitro treatment suggests good biocompatibility at higher doses of 500 μg/ml in Sw-620 cells (human colon cancer cells); however, under 30 min of thermal neutron beam irradiation leads to increased cancer cell death and a significant drop in colony-forming ability [44]. Of note, this study lacks in vivo demonstration of treatment ability.

Although the abovementioned boron-enriched nanoformulations proved to be effective boron agents overcoming the bottlenecks of conventional BSH and BPA, they still lack the element of imaging or multifunctionality. Therefore, we need to design multifunctional/theranostic boron-enriched agents to make them practically effective and economically viable boron drugs.

## Nanotheranostic Agents Meeting BNCT

Theranostic nanomedicine offers diagnosis and therapy in a single platform, enabling imaging-guided treatment of diseases while simultaneously monitoring therapeutic efficacy in real time. This approach offers significant economic benefits by improving patient outcomes, reducing the need for aggressive treatments, minimizing side effects, and imposing a relatively low economic burden on patients.

Considering this, we can see that the above boron nanosystems lack a noninvasive imaging ability for examining tumor location/size/growth and quantitative biodistribution of ^10^B-containing drugs and require an extra imaging agent. This is an additional economic burden on the patients to bear. In addition, traditional diagnostic techniques such as x-rays, ultrasound, and magnetic resonance imaging (MRI) do not respond to boron. Because of this, clinicians rely on tissue analysis through biopsies. Also, without knowing the exact location and quantity of the boron agent, neutron irradiation causes more harm than benefit. Altogether, these demand the development of noninvasive multifunctional theranostic boron-enriched nanocomposite(s) for effective BNCT application. Hence, to overcome this problem, ^11^B-based MRI techniques were used in the University of Tennessee in 1987 in Fischer rats to evaluate the pharmacokinetics of an BNCT agent for the first time [45]. At present, intravoxel incoherent motion and diffusion tensor imaging are extensively used to quantify the distribution of theranostic agents’ diffusion or perfusion into the target site based on microstructural tissue properties [46,47]. These techniques have extensively been used in predicting the radiotherapeutic response on head and neck cancers [48]. In particular, diffusion tensor imaging is an excellent modality for the diagnosis, prognosis, and prediction of treatment response for gliomas [49]. Position emission tomography (PET) and single-photon emission computed tomography are powerful tools for noninvasive imaging of pharmacological and biochemical processes in both preclinical and advanced clinical research settings.

Bifunctional boron-based NPs fashioned with F-18 offer an early pharmacokinetic assessment of patients, aiding in the subsequent treatment planning of BNCT. In addition to BNCT treatment, radiofluorine agents are Food and Drug Administration approved, exhibit high biocompatibility and less cytotoxicity, provide theranostics in a single particle, and have good dispersibility and excretion [50]. Imahori et al. [51,52] used PET to quantitively measure a radiolabeled boronated drug (^18^F–BPA) in patients with high-grade gliomas and provide its pharmacokinetics in tumor. Through this, the ^10^B in the target site could be quantified prior to neutron bombardment. Based on their influx ratios into the tumors, the duo of ^10^B with PET has a great potential to screen head and neck malignancies [53,54], oral and cervical lymph node metastasis [55], low-grade brain tumors and malignant gliomas, and recurrent malignancies [56–58]. ^18^F–BPA is not an ideal combination due to multiple reasons, including the microgram (^18^F) and milligram (BPA) dosage requirements for imaging and BNCT treatment, respectively [59]. Furthermore, head cancers usually show poor prognosis, easy recurrence, and high invasiveness. Although BNCT is effective in glioma reduction, it still faces a roadblock due to its inability to cross the blood–brain barrier (BBB); hence, it requires precise permeability targeting strategies to cross the BBB [60,61].

In addition to improved BBB permeability, to overcome undesired ^10^B leakage and achieve on-demand imaging ability, Kuthala et al. designed ^10^B-enriched (96% ^10^B) NPs (^10^BSGRF NPs) surface tailored with a fluorescein isothiocyanate (FITC)-labeled RGDK peptide (Fig. 4C). ^10^BSGRF NPs can pass through the BBB without any assistance, selectively targeting GBM sites, and deliver a high therapeutic dosage of 50.5 μg ^10^B g^−1^ cells with a good tumor-to-blood boron ratio of 2.8 and a high contrast of MRI for tracking and diagnosing the location/size/progress of brain tumor [62]. Similarly, gadolinium-157 (^157^Gd) is also a potential element for NCT, providing intrinsic T_1_-weighted MRI [63]. Furthermore, researchers have developed novel ^10^B-containing NPs using gold NPs or mesoporous silica NPs combined with Gd [58,64,65]. Fe–B (iron–boron) composite NPs were synthesized for their ability to provide tracking and quantification by MRI [66,67].

Furthermore, chemodynamic therapy (CDT) exploits Fenton or Fenton-like reactions to generate hydroxyl radicals (^•^OH) in the microenvironment of the tumor due to the mild acidity and overproduced hydrogen peroxide (H_2_O_2_) [68,69]. Researchers have designed boron-doped iron (FeB) NPs, which exhibited MRI-guided effective CDT-mediated killing of tumor cells through Fenton-like reactions. It should be kept in mind that boron doping restricted the growth of an oxide layer on NPs, making them efficient catalysts with a consistent CDT activity [70]. However, this study lacks BNCT treatment, most probably due to the lack of BNCT facility or the cost associated with it. A copolymer (fluorescent galactose-targeted glycopolymer containing *m*-carborane) was self-assembled to form micelles followed by modification with a cyanin near-infrared (NIR) dye. These self-assembled NIR micelles offer imaging-guided boron neutron capture treatment. These boron-enriched NIR micelles do not show cytotoxicity to HepG2 cells. This multifunctional platform offers fluorescence-induced tracking of NPs and provides information on when and where neutron irradiation should be used [71].

Multifunctional biocompatible gold NPs engineered with FITC (fluorescent dye), BPA, and folic acid showed high uptake by neoplastic cells due to the presence of folate receptors and in vitro optical imaging due to the presence of FITC [72]. However, reproducing such a complex formulation on commercial scale is challenging and demands simple design. Given that, dicarbolide functionalization with pendant Lewis bases leads to the formation of a stable complex with lanthanides and provides simultaneous fluorescence-imaging-guided BNCT treatment [73]. A multifunctional theranostic nanoplatform consisting of copper(II) phthalocyanine (CuPc) molecules effectively provides imaging-guided PDT. This system also leads to the amplification of microRNA (the miR-21 concentration was 0.7 fm) and a strong surface-enhanced Raman spectroscopy signal due to CuPc@HG@BN, providing early diagnosis. Due to CuPc intercalation with hairpin G-quadruplex DNA, it acted as an efficient photosensitizer for PDT with improved solubility. Both in vitro and in vivo data confirmed that this theranostic nanoplatform is ultrasensitive and selective and have high tumor accumulation (breast tumor) and a remarkable PDT outcome with no harm to normal tissues [74]. Gadolinium–boron-conjugated albumin (Gd-MID-BSA) was prepared by tagging a bovine-serum-albumin-linked maleimide-functionalized gadolinium complex and maleimide-functionalized *closo*-dodecaborate orthogonally. T_1_-weighted MRI showed that the concentration of Gd-MID-BSA in CT26-tumor-(colon cancer)-bearing mice reached the maximum level at 24 h (Fig. 4D and E), exceeding the threshold required for NCT. These results contribute to the advancement of MRI-guided NCT as a potential treatment for malignant tumors [75]. Again, in this study, the colon tumor model was a subcutaneous tumor and did not mimic the actual complexity of colon cancer.

Although preclinical data with theranostic boron nanoagents showed promising results, the toxicity rates are still relatively high. In order to accelerate the discovery of novel nanotheranostic boron drugs, we also need to focus on safety (toxicology) studies of newly designed nano-boronated materials to improve the therapeutic ratio of treatment and to decrease systemic toxicity. This can be achieved by following GMPs in preclinical studies as well.

## Nanotoxicity of Boron Nanocomposites

To advance the development of novel boron-enriched nanocomposites for BNCT, it is essential to conduct toxicological profiling of newly designed boron-based nanocomposites. This process ensures their safety and effectiveness in clinical settings by evaluating their interactions with biological systems. Additionally, performing preclinical nanotoxicological assessments of these agents according to GMPs will further improve patient care, reduce the risk of costly treatment failures, and increase the likelihood of successful clinical translation.

**Fig. 5.**
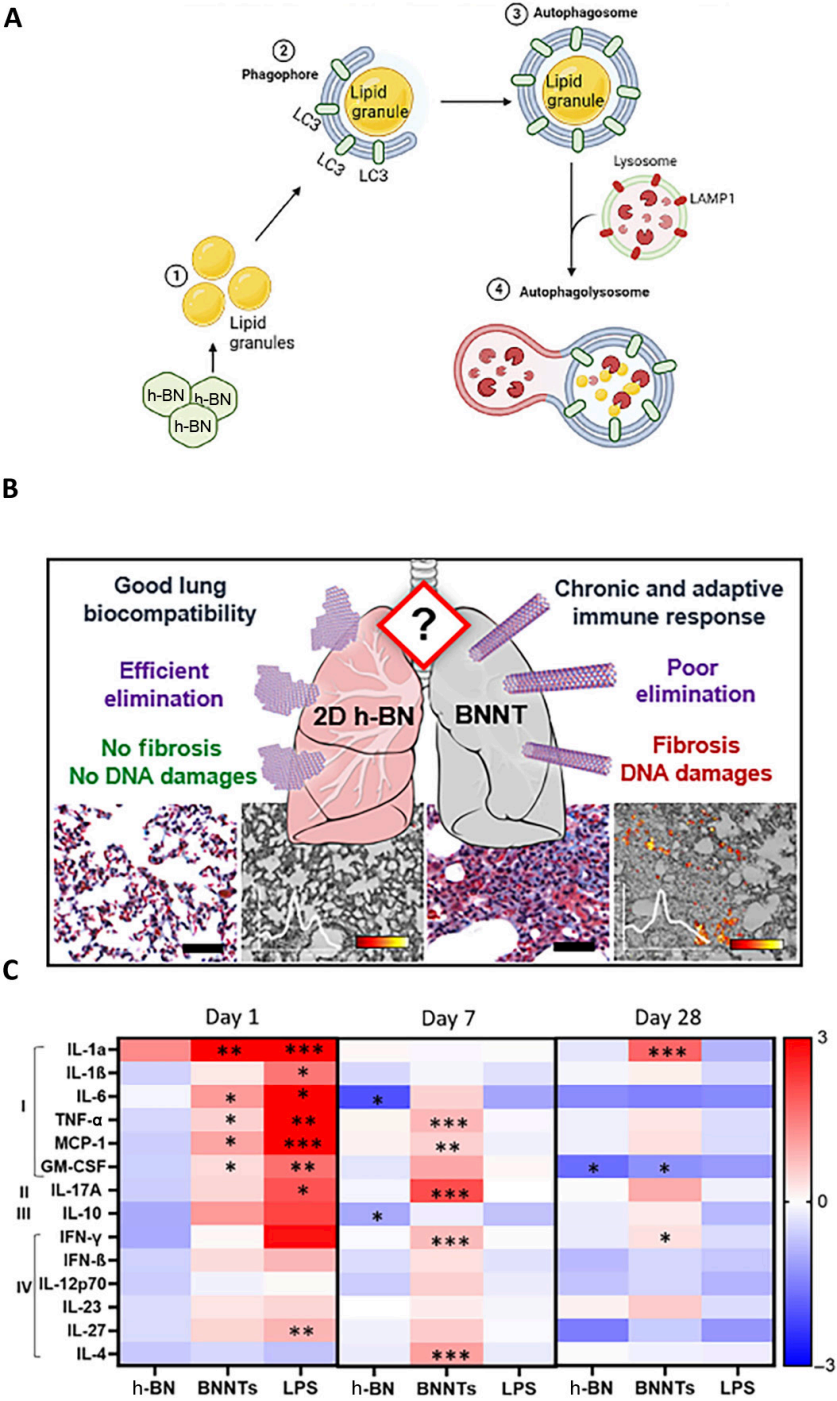
(A) Hexagonal boron nitride (h-BN)-triggered cellular autophagy involves its uptake by alveolar lung cells making lipid granules, leading to autophagy due to overproduction of cellular lipid granules. Reproduced with permission from Ref. [84]. (B) Pulmonary toxicity of h-BN and boron nitride nanotubes (BNNTs). Scale bars: 20 μm. (C) Evaluation of inflammatory markers levels in lungs by multiplex enzyme-linked immunosorbent assay (ELISA) as log_2_ fold change. (I) Acute inflammation; (II) late pro-inflammatory response; (III) anti-inflammatory response; (IV) adaptive immune activation. Reproduced with permission from Ref. [86]. *P < 0.05; **P < 0.01; ***P < 0.001. LC3, microtubule-associated protein 1A/1B-light chain 3; LAMP1, lysosomal associated membrane protein 1; 2D, 2-dimensional; IL, interleukin; TNF-α, tumor necrosis factor alpha; MCP-1, monocyte chemoattractant protein-1; GM-CSF, granulocyte-macrophage colony-stimulating factor; IFN, interferon; LPS, lipopolysaccharide.

Hexagonal boron nitride (h-BN) and boron nitride nanotubes (BNNTs) show high similarity with carbon-based nanomaterials due to molecular organization and the presence of an aromatic condensed structure [76]. Therefore, like carbon nanomaterials, these boron nanomaterials are relatively highly biocompatible and showed a relatively high safety profile. However, their extensive use in electronics and medicine could also lead to their unintentional and intentional inhalation during production, processing, and inappropriate handling, leading to various adverse effects. For example, BNNTs and multiwall carbon nanotubes produce similar toxic outcomes in vivo and in vitro [77], as 1-month exposure to 50 wt% pure BNNTs leads to lung inflammation, indicated by the high infiltration of granulocytes (Fig. 5A) and lymphocytes [78]. In particular, BNNTs cause chronic lung inflammation due to permanent tissue damage (DNA damage and fibrosis) attributed to delayed clearance from lungs. Of course, these damaging effects become more prominent with increasing aspect ratio and mechanical strength [79,80]. Usually, h-BN and BNNTs showed adverse effects after 24-h exposure; therefore, studies limited to 24-h toxicity profiling could not be reliable. They both (h-BN and BNNTs) cause significant release of pro-inflammatory cytokines (interleukin-1β and granulocyte-macrophage colony-stimulating factor). Conversely, BNNTs are considered comparatively more toxic than h-BN (100 μg/ml) due to their ability to stimulate the NLRP3 (NLR family pyrin domain containing 3) inflammasome even at a very low dose of 25 μg/ml [81]. Due to their morphology-mediated toxicity, BNNTs fall into the fibrous material pathology and therefore induce adverse phagocytosis in alveolar macrophages [82,83]. h-BN distorts cell (A549) membrane integrity (Fig. 5B and C), epithelial barrier integrity, interleukin-8 cytokine production, or reactive oxygen species generation in a dose-dependent way (1.5 and 10 μg/cm^2^), affecting lipid metabolism and autophagy by hijacking normal homeostasis. However, this course of homeostasis dysregulation leads to reduced lysosomal function, resulting in accumulation of autophagosomes the cells [84,85]. A recent study also confirmed that oropharyngeal aspiration of BNNTs activates acute and chronic inflammation and innate and adaptive immunodeficiency-mediated granulomatous inflammation. A possible reason is that BNNTs are not eliminated from the lungs and they bioaccumulate into larger agglomerates, ultimately increasing their retention time in the lungs. Due to their higher biopersistence and chronic activation of immune response, BNNTs cause pulmonary fibrosis and DNA double strand breaks, leading to long-term chronic (Fig. 5B and C) pulmonary diseases [85,86].

Boron nitride nanospheres (BNNSs) have applications in polymer filling and biomedicine [87]. BNNSs coated with 10-nm Fe_3_O_4_ NPs, named Fe_3_O_4_@BNNS), showed concentration-dependent in vitro toxicity (MCF-7, MCF-10, and HeLa cell lines) in 24-h exposure conditions. This study showed that Fe_3_O_4_@BNNS nanocomposites are nontoxic to cancer cell lines at lower concentrations; however, they exhibit notable toxicity at higher concentrations. This study did not elaborate on the mechanism of toxicity, and a possible reason for toxicity at higher concentrations might be the possible peroxidase enzyme mimic activity arising due to the presence of Fe_3_O_4_ NPs, which was not explained in this study [88]. BCDs with an average diameter of 4 nm with strong fluorescence in the blue spectrum with 33% quantum yield (photoluminescence quantum yields) were synthesized. Evaluation of the 24-h in vitro toxicity testing of BCDs with HeLa cells showed that BCDs had low toxicity. Furthermore, the long-term (31-d) in vivo toxicity of BCDs with a dose of 20 mg/kg in female mice inflicted liver and kidney toxicity. However, BCDs were completely biocompatible for pancreas; no change in physical behavior and body weight was observed, and they did not cause any death [89]. Given the liver and kidney toxicities will limit the clinical use of BCDs specifically and boron-containing NPs in general. Researchers effectively used the chaotropic effect to overcome the biotoxicity of boron-enriched compounds, such as the highly soluble cationic dye malachite green (MG). The chaotropic effect is a noncovalent effect providing a driving force for modular assembly of flexible materials and impacting the biocompatibility and toxicity of the materials [90,91]. Researchers used self-assembly between *closo*-dodecaborate clusters ([B_12_H_12_]^2−^) and MG, forming an insoluble cubic-shaped supramolecular complex (B_12_–MG) [92], transforming MG into a completely biocompatible one. Furthermore, h-BNs with different average sizes of 40 to 270 nm did not cause any adverse effects on silkworm, leading to 8% death after 96-h incubation. However, genes involved in midgut functions were altered and require detailed information on that [93]. BNNPs caused subacute toxicity in mice with repeated administration of increasing dosage from 26 to 135 mg/kg, once every other day for 14 d. However, the subacute toxicity of BNNPs can be reduced by coating them with biocompatible erythrocyte membranes [94].

In short, although early findings with BNCT suggest promising potential, the relatively high toxicity rates remain a significant challenge, limiting its broader clinical applications. To address this issue, further research is urgently required to develop more selective and effective boronated (nano)composites. These advancements are expected to not only improve the therapeutic ratio but also minimize the adverse effects of boron-based nanocomposites, ultimately making BNCT a safer and more viable treatment option for patients.

## Systems Biology Approach and BNCT

Systems biology is an interdisciplinary arena that uses computational and mathematical techniques to model complicated biological systems [95]. It involves the combined and consolidative use of various scientific domains: engineering, biology, computer science, bioinformatics, chemistry, physics, and more. In systems biology, networks are used to combine omics data, representing the convoluted communications that occur within biological structures at distinctive levels such as genes, transcripts, proteins, and metabolic pathways. This tactic helps us understand the global molecular mechanisms and biological functions of these systems [96–99]. For instance, proteomic analysis of extracellular vehicles (EVs) isolated from SAS (oral cancer cell line) cultures treated with ^10^B-BPA and different doses of neutron irradiation elaborated that BPA^−^ and BPA^+^ affect EV-derived proteins involved in apoptosis, DNA repair, and inflammatory response. For example, Krueppel-like factor 11, serpin family E member 1, and serpin family F member 2 were up-regulated in BPA^+^, while DNA polymerase epsilon 2, accessory subunit and serpin family C member 1 were up-regulated in BPA^−^, indicating the systemic effects of boron infusion and/or neutron irradiation. Proteomic analysis of patient-derived EVs shed light on circulating signals linked with early cancer detection and radiotherapy [100].

## Boron Nanocomposites for Comorbidity Treatment

Maximizing the effectiveness of BNCT for cancer treatment using multifunctional boron-based nanocomposites remains a complex yet promising area of research, particularly for patients with comorbid conditions, such as diabetes, cardiovascular disorders, Alzheimer’s disease, and other chronic ailments. These conditions often share underlying pathological mechanisms, including oxidative stress, inflammation, and metabolic dysregulation, and accelerate cancer progression and reduce BNCT efficacy. Developing boron-based nanocomposites with multifunctional properties—such as anti-inflammatory, antioxidant, and targeted therapeutic functionalities—offers a potential solution to address these challenges. Such strategies can concurrently mitigate the impact of comorbidities and enhance the efficiency of BNCT, ultimately improving patient outcomes. Futhermore, boron-containing nanomaterials’ unique physicochemical characteristics—such as high resistance to oxidation, stable electrical properties, and hydrophobicity—extend their potential applications in gene and drug delivery [101], tissue regeneration [102], photothermal treatment [103], enzyme inhibitors [104], antiviral treatment [105], and other biomedical and technological fields [106–108].

**Fig. 6.**
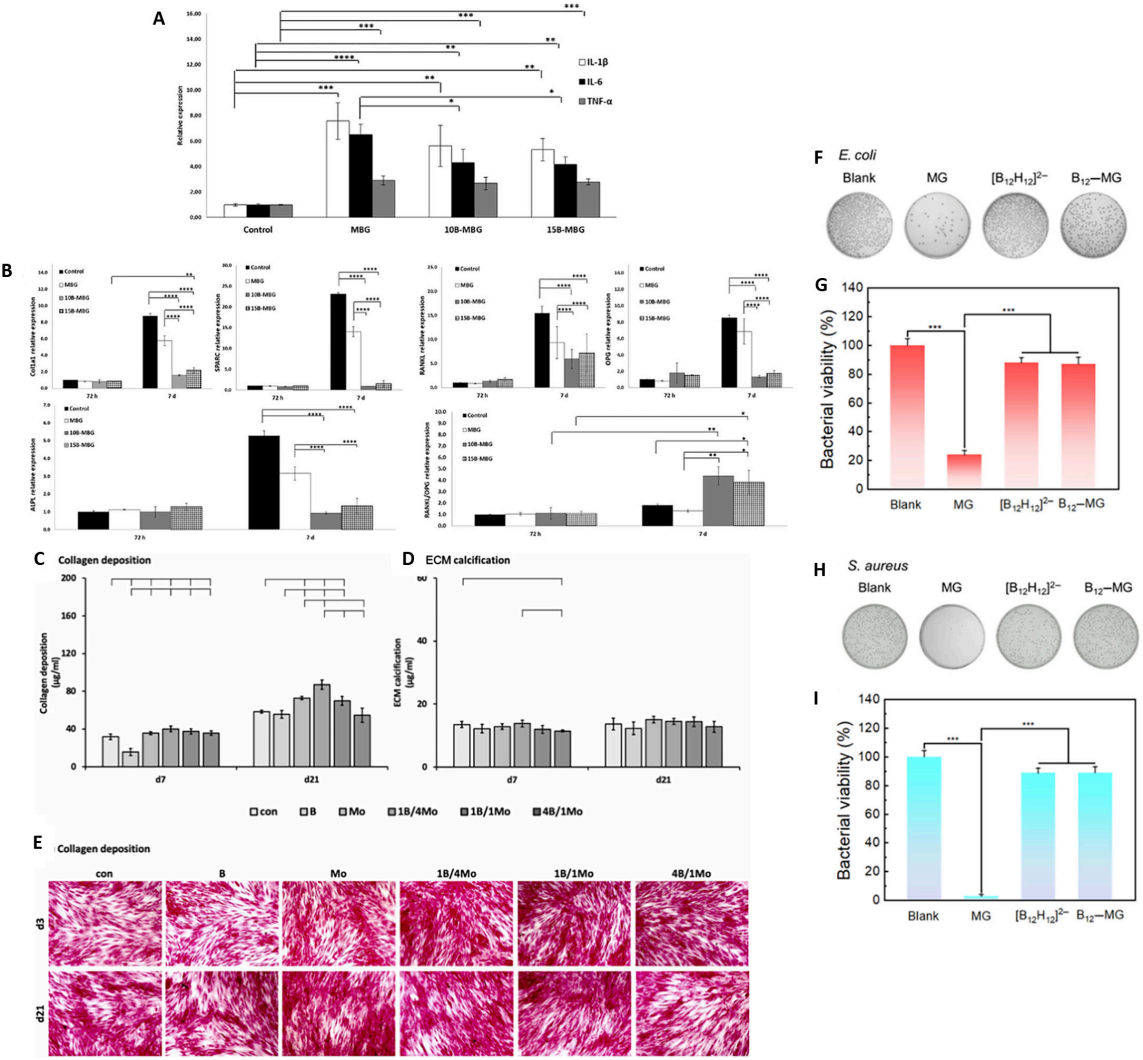
(A) Reverse transcription quantitative real-time polymerase (RT-qPCR) analysis of pro-inflammatory genes IL-1β, IL-6, and TNF-α in macrophages incubated with mesoporous bioactive glass (MBG) and boron-containing MBG for 4 h at 1 mg ml^−1^. (B) RTqPCR analysis of pro-osteogenic genes COL1A1, ALPL, SPARC, RANKL, OPG, and RANKL/OPG in osteoblast-like SaOS2 cells incubated with MBG and boron-containing MBG at the concentration of 1 mg mL−1 for 72 h and 7 days. Reproduced with permission from Ref. [102]. *P ≤ 0.05; **P ≤ 0.01; ***P ≤ 0.001; ****P ≤ 0.0001. (C to E) Effects on collagen deposition and extracellular matrix (ECM) calcification. Bone-marrow-derived mesenchymal stromal cells (BMSCs) were cultured for 7 and 21 d in the presence of B, Mo, and their combinations at a total concentration of 2.0 mM. Reproduced with permission from Ref. [112]. Antibacterial activity of B_2_O_3_–ZnO NPs on (F) *Escherichia coli* and (H) *Staphylococcus aureus* agar plates treated by different materials at 1 mM. Bacterial survival of (G) *E. coli* and (I) *S. aureus*. Reproduced with permission from Ref. [92]. ***P < 0.001. 10B-MBG, 5.8 mol% B_2_O_3_–mesoporous bioactive glass nanoparticles; 15B-MBG, 6.5 mol% B_2_O_3_–mesoporous bioactive glass nanoparticles; Col1A, collagen, type I, alpha 1; ALPL, alkaline phosphatase, biomineralization associated; SPARC, secreted protein acidic and cysteine rich; RANKL, receptor activator of NF-κB (RANK) ligand; OPG, osteoprotegerin; d7, day 7; d21, day 21; d3, day 3; con, control; MG, malachite green.

For instance, interaction between a BNNT layer and mesenchymal stem cells (MSCs) evidenced the strong adsorption of MSCs and their normal growth, proving the biocompatibility of BNNTs. In particular, BNNTs synergize alkaline phosphatase activity as an early marker of osteoblasts and alkaline phosphatase/total protein and osteocalcin as a late marker of osteogenic differentiation, confirming that BNNTs enhance the osteogenesis of MSCs. However, traces of released boron and the stress on cells due to the fibrous morphology of BNNTs further promote osteogenic differentiation of MSCs, leading to bone regeneration [109]. Similarly, boron-containing mesoporous bioactive glass NPs regulate inflammatory response (down-regulated pro-inflammatory genes) for bone regeneration due to the elevated activity of macrophages and interrupt osteogenic differentiation into osteoblasts [102] (Fig. 6A and B). In addition, encapsulated boron within scaffolds promoted the proliferation and differentiation of preosteoclast cells [110]. Moreover, the potential role of boron in tissue engineering is due to enhanced myotube formation and myoblast differentiation [111]. For instance, the combination of boron with molybdenum (Mo) leads to enhanced vascularization and bone regeneration [112]. As shown in Fig. 6C and D, BNNPs, tailored with gum arabic, demonstrated cytocompatibility and supported cell attachment, proliferation, and differentiation into adipocytes and osteocytes [113,114]. In another study, BNNT incorporation into gelatin–glucose scaffolds showed no influence on cell viability, decreased the scaffold degradation rate, and improved cell attachment and proliferation compared to only-gelatin scaffolds [115]. While BNNTs improved stem cell differentiation, it is important to note that they have cytotoxic effects and cause DNA damage at certain concentrations, possibly due to increased reactive oxygen species levels [116]. Nonetheless, the potential cytotoxicity and differentiation-affecting attributes of boron-enriched nanomaterials pose risks that need to be carefully considered in the context of stem cell technology and cell-based therapies [117]. Furthermore, their potential applications extend beyond stem cell differentiation to include safe drug delivery, tissue engineering, and enhancing the mechanical properties of scaffolds for bone tissue engineering.

In addition to anticancer activity, gum-arabic-coated bimetallic B_2_O_3_–ZnO NPs proved promising against *Escherichia coli*, *Pseudomonas aeruginosa*, *Bacillus subtilis*, and *Staphylococcus aureus* at 125, 62.5, 125, and 62.5 mg ml^−1^, respectively. In addition, B_2_O_3_–ZnO NPs showed antifungal activity against *Candida albicans* (a unicellular fungi) at a 62.5 mg ml^−1^ concentration. This antibacterial and antifungal activity is due to the intrinsic antioxidant activity (IC_50_ was 102.6 mg ml^−1^) due to the generation of free radicals by B_2_O_3_–ZnO NPs [118]. This could be beneficial in treatment of complex pathological conditions in which cross-kingdom biofilm formation leads to restricted or hard-to-treat diseases.

The involvement of protein and polypeptide self-assembly in various degenerative diseases, including Alzheimer’s disease, Parkinson’s disease, and Huntington’s disease, has garnered significant attention in recent years [119,120]. Misfolding of these proteins and peptides, despite the lack of structural or functional similarity, results in the formation of amyloid fibrils T [119,121]. These fibrils adopt a characteristic cross β-sheet structure, where protofibril units composed of parallel β-strands are assembled laterally to form larger fibers [121–123]. Amyloid deposits are present in over 90% of patients with type 2 diabetes (T2D) [124]. The islet amyloid found in the pancreas of T2D patients consists of a 37-residue-long polypeptide called human islet amyloid polypeptide (hIAPP). hIAPP is a neuroendocrine hormone with the sequence K​1CN​TAT​CAT​Q10​RLA​NFL​VHS​S20​NNF​GAI​LSS​T30NVGSNTY37. Costored and cosecreted with insulin in pancreatic β-cells, hIAPP plays a role in delaying gastric emptying and inhibiting glucagon secretion, thereby contributing to glucose regulation [125]. BNNPs have emerged as promising antiamyloid agents due to their low systemic toxicity. Studies demonstrated that BNNPs can inhibit the dimerization of hIAPP, a process linked to the pathogenesis of T2D. These NPs (BNNPs) prevent hIAPP misfolding into β-sheet-rich aggregates with varying curvatures, inhibiting the progression and severity of T2D. For example, BNNP hydrophobicity increases from (5,5)BN nanotubes to BN nanosheets, and their interaction shifts from the N-terminal to the amyloid-prone C-terminal of hIAPP. For instance, (5,5)BNNTs primarily bind to the Gln10-Phe23 region, (10,10)BN nanotubes to the Asn14–Leu27 region, and BN nanosheets to the Asn21-Val32 region [126]. Hydrophobic interactions and aromatic stacking contribute to the binding affinity between hIAPP and BNNPs, with the flat surface of nanosheets showing stronger affinity compared to nanotubes. Additionally, BNNPs disassemble dimerized hIAPP fibrils, with this effect being more pronounced in the (5,5) nanotube and nanosheet morphologies.

In conclusion, targeting the comorbidities in cancer patients could simultaneously mitigate the progression of chronic diseases and significantly improve the effectiveness of BNCT for cancer treatment. In addition, providing solutions for various diseases through single boron-enriched nanomaterials makes them an ideal candidate to explore and reach clinical settings.

## Summary and Prospects

BNCT has demonstrated remarkable advancements, but its widespread clinical implementation faces multiple challenges. These include technical hurdles, regulatory barriers, economic constraints, and patient-related factors. Despite these obstacles, ongoing research in boron-rich nanomaterials and novel neutron sources continues to push BNCT toward broader biomedical applications. The following points outline key developments, existing challenges, and future directions:

• The development of boron-rich nanocomposites with multifunctional properties—such as controlled release, environmental responsiveness, and strong therapeutic effects—remains central to improving BNCT. These materials require advanced design strategies that integrate computational methods, combinatorial experimental techniques, and machine learning. These approaches will expedite the discovery of optimized boron-rich theranostic nanomaterials while reducing time and resource consumption, thereby enhancing the overall effectiveness of BNCT agents.
• Modern material development must adhere to environmental, social, and governance criteria. As sustainability becomes a primary focus, new boron-rich theranostic nanomaterials must not only meet therapeutic goals but also align with these ethical and environmental standards. This shift in focus requires innovative approaches to design, ensuring that the materials used are both effective in treatment and compliant with global sustainability frameworks.
• With the rise of accelerator-based BNCT facilities, addressing logistical and economic challenges is essential. Cost-effective treatments are necessary to ensure wider access to BNCT. This can be achieved through the development of more affordable boron delivery agents and by improving tumor-targeting strategies, such as incorporating nanomaterials with high tumor uptake and targeted delivery systems.
• The design of boron-containing compounds must focus on enhancing pharmacokinetics and stability and minimizing toxicity. Achieving these improvements requires overcoming the inherent inertness of boron compounds, which limits their functionalization for targeted delivery. Research efforts must therefore concentrate on developing more sophisticated, stimuli-responsive systems capable of precise drug delivery and controlled release at specific sites within the body.
• Successful clinical translation of BNCT will require the integration of systems biology approaches with preclinical studies. Combining BNCT agents with biological and immunological models can help clarify their mechanisms of action and improve therapeutic outcomes. Moreover, “omics” technologies—including genomics, proteomics, and metabolomics—will provide deeper insights into BNCT’s effects at the molecular level, aiding in the identification of biomarkers for disease stratification and personalized treatments.
• Comprehensive toxicity evaluations of BNCT agents are crucial for ensuring their safe and effective clinical application. Additionally, stringent quality control measures for accelerator-based BNCT reactors must be implemented to maintain safety standards. Developing predictive models, such as organ-on-chip systems or human-derived organoids, will also be key to assessing nanomaterial safety and optimizing treatment regimens.
• The future of BNCT lies in the development of multitargeted boron-based nanomaterials capable of exerting synergistic therapeutic effects. Such materials, when integrated into intelligent drug delivery systems, could respond to various stimuli—such as pH, temperature, or enzyme activity—ensuring more precise and effective treatments. Further research is needed to refine these systems for enhanced specificity and improved patient outcomes.
• As the complexity of boron-based nanomaterials increases, so do the regulatory requirements for their approval. Ensuring reproducibility, safety, and biocompatibility is an essential step in transitioning these materials from laboratory research to clinical practice. Regulatory frameworks must evolve to accommodate the growing complexity of these nanomaterials, and innovative methodologies such as microphysiological systems and human-derived induced pluripotent stem cells will be critical for predicting their behavior in humans.
• Boron-rich nanomaterials potential extends beyond oncology, with emerging applications in treating complex diseases such as neurodegenerative disorders and infectious diseases. The ability to design multifunctional boron nanomaterials opens new avenues for various pathological conditions. These applications could further enhance the versatility of boron-rich agents as a broad-spectrum therapeutic modality in biomedicine.
• Establishing networks between BNCT centers using various neutron sources is critical for optimizing neutron dosimetry and harmonizing treatment protocols. These collaborations, however, are logistically demanding and require sustained effort to maintain coordination across multiple disciplines and facilities.

In conclusion, while BNCT faces several challenges, the continued development of boron-rich nanomaterials, innovative neutron source technologies, and collaborative research efforts offer promising avenues for overcoming these obstacles. By addressing economic, regulatory, and technical challenges, BNCT holds the potential to become a transformative therapeutic strategy not only for cancer but for a wide range of diseases.

## Data Availability

Data sharing is not applicable for this article as no datasets were generated or analyzed during the current study.
